# Computed tomography accuracy and features in detecting open globe injuries in patients with ocular trauma: a systematic review and meta-analysis

**DOI:** 10.25122/jml-2024-0353

**Published:** 2025-01

**Authors:** Ghada Aljuhani, Nahla Aljuhani

**Affiliations:** 1 Ophthalmology Department, Saudi Commission for Health Specialties, Madinah, Saudi Arabia; 2 Radiology Department, King Fahad Hospital, Madinah, Saudi Arabia

**Keywords:** open globe, rupture globe, computed tomography, ocular trauma

## Abstract

Open globe injuries (OGIs) can have devastating impacts on patients’ lives. Early detection of OGIs is crucial for improving outcomes, as any delay in treatment can result in significant consequences. Radiological imaging, particularly computed tomography (CT), aids ophthalmologists in diagnosing this condition, especially in challenging cases. This literature review aimed to evaluate the accuracy of CT scans in identifying features of OGIs based on current evidence. This systematic review adhered to the 2020 Preferred Reporting Items for Systematic Reviews and Meta-Analyses (PRISMA) protocol. PubMed and EBSCOhost databases were searched using the keywords 'rupture globe' or 'open globe' and 'computed tomography' or 'CT'. Articles published in English between 1990 and 2023 were considered for inclusion, whereas review articles were excluded from the analysis. The initial search yielded 169 studies, and nine studies were included in the final screening. This review encompassed 917 eyes. The pooled specificity of the CT scan in detecting OGIs among patients with ocular trauma was 0.94 with 95% CI (0.92–0.96), and the pooled sensitivity was 0.77 with 95% CI (0.72–0.81). The area under the curve was 0.91, indicating the excellent ability of the CT scan to detect open globe injury. CT scans demonstrated high diagnostic accuracy for detecting OGI. While CT is highly effective in identifying ocular trauma, careful interpretations remain essential. Additional studies with larger sample sizes are recommended to refine its diagnostic role further.

## INTRODUCTION

Open globe injury (OGI) is a severe ocular condition with lasting effects on patients and healthcare systems [1,2]. In the United States, OGI occurs at a rate of 4.49 per 100,000 individuals [2]. In Saudi Arabia, the prevalence of OGI among pediatric patients with ocular trauma is 72% [3], whereas in adults, it is 37.5% [4]. According to a study by Naif Alali *et al*. [5] in Saudi Arabia, blunt trauma accounted for 42.9% of OGI cases, with males being the most affected. The final visual outcome was poor in 60% of cases at the last follow-up visit [5].

Ocular trauma encompasses closed and open injuries, with the latter characterized by a full-thickness wound at the scleral or corneal level [2]. The classification of OGIs considers many factors, including the mechanism of injury, associated tissue damage, and the affected area. Based on anatomical zones, OGIs are classified as follows: Zone I, limited to the cornea and limbus; Zone II, extending up to 5 mm posterior to the limbus; and Zone III, involving injuries beyond 5 mm behind the limbus [6].

During eye trauma evaluation, priority should be given to excluding severe and life-threatening conditions like severe head trauma [7]. Subsequently, identifying vision-threatening ophthalmological injuries such as ruptured globes is crucial. Additionally, recognizing any other ocular injuries associated with open globes, such as endophthalmitis and intraocular or intraorbital foreign bodies (IOFBs), is important [8,9].

In cases with a history of severe eye trauma and an OGI, the eye examination should be conducted with minimal tissue manipulation to reduce the risk of eye structures being extruded through the full-thickness laceration [10]. In certain situations, ruling out OGI can be challenging due to uncooperative patients (such as children or patients with developmental disabilities), extensive facial injury and eyelid swelling, or instances where a full-thickness scleral laceration is hidden under extensive subconjunctival hemorrhage [11,12].

Computed tomography (CT) is a readily available and rapid radiological modality commonly utilized in cases of ocular and orbital trauma, including orbital fractures and the detection of IOFBs [10,13]. Unlike other imaging modalities, CT scans for orbital or ocular trauma do not typically require intravenous contrast agents, enhancing their accessibility and efficiency [14]. The diagnostic accuracy of CT in detecting open globe injuries varies, with reported sensitivity ranging from 73% to 94% [11,15-22].

This variability highlights the need for a systematic review to better assess the reliability of CT in diagnosing OGI. Previous research has offered valuable insights into CT scan sensitivity and specificity in detecting OGI, but it needs the comprehensive analysis that a systematic review provides. Our study addresses this gap by systematically collecting and analyzing existing evidence, offering a more comprehensive understanding of the clinical role of the CT scan in managing OGI.

## MATERIAL AND METHODS

### Search strategy

A systematic search was conducted in PubMed and EBSCO databases in December 2023. The keywords used for the search included 'rupture globe' OR 'open globe' AND 'computed tomography' OR 'CT'. Articles published in English between 1990 and 2023 were included in the search. This review followed the Preferred Reporting Items for Systematic Reviews and Meta-Analyses (PRISMA) 2020 guidelines.

The review was not registered, and no protocol was prepared.

### Selection criteria

**Figure 1:**
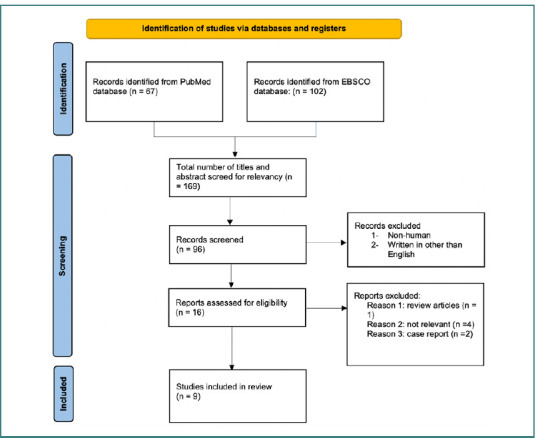
Preferred Reporting Items for Systematic Reviews and Meta-Analyses (PRISMA) flowchart

The inclusion criteria for this study encompassed research assessing CT scan accuracy in detecting open globe injuries, published in English between January 1990 and December 2023. Eligible study designs included cross-sectional studies, case reports, clinical randomized trials, and research involving humans. Exclusion criteria involved review articles, publications in languages other than English, and studies lacking accuracy parameter measurements. Initially, 169 studies were reviewed. A detailed flowchart of this process is provided in Figure 1.

### Screening and data extraction

Two independent reviewers conducted the database search and screened abstracts for relevance. Full-text articles meeting the eligibility criteria underwent further assessment. Data extraction was conducted using an Excel spreadsheet by two independent reviewers, collecting information on the first author’s name, publication date, country, study design, sample size, age, and gender. Extracted outcome measures included positive predictive value (PPV), negative predictive value (NPV), sensitivity, specificity, and accuracy of CT scans in detecting OGIs. These data were obtained from each study included. Studies were not grouped based on specific criteria; all relevant studies meeting the inclusion criteria were incorporated collectively.

### Quality assessment

Two independent researchers reviewed the articles for risk of bias using the Quality Assessment of Diagnostic Accuracy Studies-2 (QUADAS-2) tool, which evaluates bias in diagnostic accuracy studies [23]. The assessment focused on two key domains: risk of bias and applicability, each incorporating patient selection, index test, and reference standard. The Oxford Level of Evidence Hierarchy was used to assess the quality of evidence.

### Statistical analysis

We conducted the meta-analysis using Meta-DiSc version 1.4, which was designed for accuracy meta-analysis. Additional statistical analyses were performed using MedCalc version 22.016.

## RESULTS

### Risk assessment

We found four studies with a high risk of bias in patient selection, raising concerns regarding the generalizability of the study population. Additionally, one study was flagged for a high risk of bias in the index test, potentially impacting the diagnostic accuracy of the utilized tool. Furthermore, one study had an unclear risk of bias in patient selection, indicating a lack of clarity in the methodology documentation. Table 1 shows a detailed description of the quality of the included studies.

**Table 1:** Risk of bias in included studies based on QUADAS-2 assessment

Study	Risk of bias				Applicability concerns		
	Patient selection	Index test	Refrence statndard	Flow and timing	Patient selection	Index test	Refrence statndard
1. Wei-Hsin Yuan *et al.*	⇑	⇑	⇑	⇑	⇑	⇑	⇑
2. Chieh Chou *et al.*	⇑	⇓	⇑	⇑	⇑	⇓	⇑
3. Seong Yun Kim *et al.*	⇓	⇑	⇑	⇑	⇓	⇑	⇑
4. Khaled Gad *et al.*	⇑	⇑	⇑	⇑	⇑	⇑	⇑
5. D P Joseph *et al.*	⇔	⇑	⇑	⇑	⇔	⇑	⇑
6. Amir Arabi *et al.*	⇓	⇑	⇑	⇑	⇓	⇑	⇑
7. Eric L Crowell *et al.*	⇑	⇑	⇑	⇑	⇑	⇑	⇑
8. Mark L Arey *et al.*	⇓	⇑	⇑	⇑	⇓	⇑	⇑
9. P Hoffstetter *et al.*	⇓	⇑	⇑	⇑	⇓	⇑	⇑

Low risk ⇑, High risk ⇓, Unclear ⇔

### Study characteristics

A total of 883 patients (917 eyes) were included in the analysis, with 40% diagnosed with OGIs. Our analysis comprised nine observational studies between 2000 and 2021, predominantly conducted in the USA (55.55% of studies). The mean age of participants was 38.72 years, with 478 male and 141 female patients. However, two studies lacked age and sex data. Additional details on study characteristics are provided in Tables 2 and 3.

**Table 2:** Study characteristics

Author	Publication date	Study design	Level of evidence	Country	Sample size	Gender	Mean age
Wei-Hsin Yuan [15]	2014	Observational study	2	Taiwan	75 patients (76 eyes) 33 were ruptured globe	M = 56 F = 19	45.1 years
Chieh Chou [16]	2016	Observational study	2	Taiwan	136 patients(136 eyes)32 of them were ruptured globe	M = 99 F = 37	41
Seong Yun Kim [17]	2010	Observational study	2	Korea	28 with open globe and 28 controls	Control M: F = 20:8 Open globe 25:3	40 for control43 for open globe cases
Khaled Gad [18]	2017	Observational study	3	USA	122 patients(122 eyes)47 were open globe	NA	NA
D P Joseph [19]	2000	Observational study	2	USA	142 patients(142 eyes)80 were open globes	NA	NA
Amir Arabi [20]	2021	Observational study	2	USA	133 patients(133 eyes)61 with open globe	M = 115 F = 18	39
Eric L Crowell [21]	2017	Observational study	3	USA	114 patients (145 eyes) of which 35 eyes with open globe	M = 83 F = 31	39.5
Mark L Arey [22]	2007	Observational study	2	USA	46 patients of(48 eyes)34 were open globe	M = 38 F = 8	36
P Hoffstetter [11]	2010	Observational study	3	Germen	59 patients (59 eyes)17 with open globe	M = 42 F = 17	29

M, male; F, female; NA, not applicable where the data is not mentioned in the original article.

**Table 3:** Diagnostic performance of CT scans

Author	Sensitivity	Specificity	PDV	NPV	Kappa (Inter observer agreement)	Accuracy	Interpretation conducted by
Wei-Hsin Yuan [15]	76%	85%	80%	82%	0.63–0.96	81%	2 neuroradiologists
Chieh Chou [16]	75%	94.2%	80%	92%	NA	89.7%	1 radiologist
Seong Yun Kim [17]	92%	85%	86%	92%	0.64	89%	2 radiologists
Khaled Gad [18]	87.2%	97.3%	95.4%	92.41%	0.876 (0.787–0.965)	94%	2 neuroradiologists
D P Joseph [19]	75%	93%	95%	74.4%	0.70	83%	2 neuroradiologist 1 ophthalmologist
Amir Arabi [20]	79% for combing both axial and coronal cut	93% for combing both axial and coronal cut	91% for combing both axial and coronal cut	84% for combing both axial and coronal cut	0.96 and 0.95 for the axial and coronal plane, respectively	87%	1 ophthalmologist and 1 neuro-radiologist
Eric L Crowell [21]	51% for neuroradiologist 71% for ophthalmologists.	100% for neuroradiologist 97.5% for ophthalmologists	100% for neuroradiologist 94.5% for ophthalmologists	82% for neuroradiologist 89% for ophthalmologists	between the neuroradiologist and ophthalmologist (0.71) and between ophthalmologists (0.88)	89%	1 neuroradiologist vs 2 ophthalmologists.
Mark L Arey [22]	71%	79%	89%	52%	NA	73%	2 neuroradiologists and 1 ophthalmologist
P Hoffstetter [11]	70%	98%	92%	89%	0.89–0.96	90%	4 radiologists

NA, not applicable where the data is not mentioned in the original article

**Figure 2:**
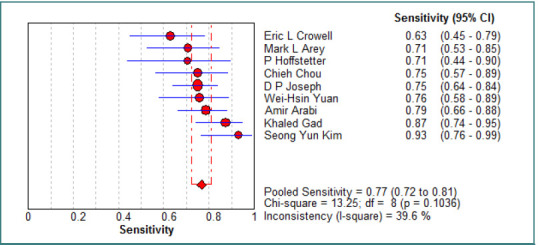
Forest plot of the sensitivity of the studies included

**Figure 3:**
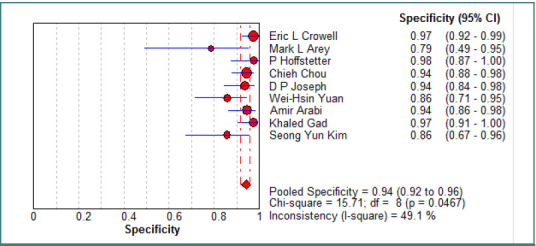
Forest plot of the specificity of the studies included

**Figure 4:**
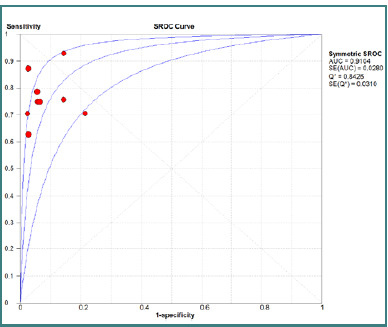
Summary receiver operating characteristic plot

The pooled specificity of CT scans for detecting OGIs was 0.94 (95% CI, 0.92–0.96), with an I^2^ of 49% (*P* = 0.04), indicating statistically significant heterogeneity among studies. The pooled sensitivity was 0.77 (95% CI, 0.72–0.81), with an I^2^ of 39.6% (*P* = 0.10), suggesting statistically insignificant heterogeneity across the included studies. Further details on sensitivity and specificity forest plots are available in Figures 2 and 3. A summary receiver operating characteristic (SROC) curve analysis (Figure 4) yielded an area under the curve (AUC) of 0.91 (SE: 0.02), indicating excellent diagnostic performance (AUC > 0.9 suggests strong discrimination between patients with and without OGIs). The Q value was 0.84, suggesting moderate heterogeneity.

### Common CT findings in open globe injuries

**Figure 5:**
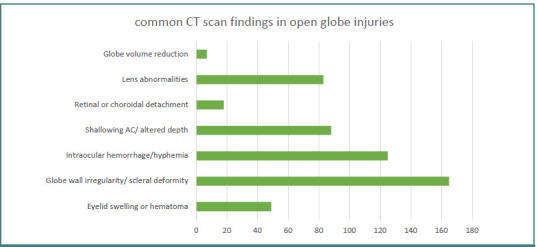
Common computed tomography signs in open globe cases

Six studies [11,15,18-20,22] reported the most common CT features associated with OGIs. The most frequently observed finding was globe wall irregularity or scleral deformity, followed by intraocular hemorrhage. A summary of CT findings is presented in Figure 5.

### Most sensitive CT signs in open globe injuries

Five of the nine studies [15,17,18,21,22] mentioned the most sensitive CT findings for detecting OGIs. Globe deformity or wall irregularity was identified in most studies as the most sensitive sign on CT scans for open globe injury. Table 4 shows the frequency of each sign across various studies.

**Table 4:** Frequency of the most sensitive CT scan features in detecting OGIs

CT scan feature	Frequency among most sensitive sings
Globe deformity / wall irregularity	4/5
Shallow AC	3/5
Lens dislocation	3/5
Intraocular hemorrhage	3/5
IOFB	2/5
Intraocular gas	1/5
Volume loss of the globe	2/5
Retinal detachment	1/5

### Anterior chamber shallowing as an indicator of OGI

In a study by Khaled Gad *et al*. [18] involving 122 patients, 47 had a ruptured globe. A CT scan diagnosed 39 patients with ruptured globes, and a shallow AC had 89.2% sensitivity and 87.1% specificity in detecting an OG, predicting it in 39 out of 47 cases. Similar findings were observed for AC density (hyphema), predicting an OG in 41 out of 47 cases. Another study by Seong Yun Kim *et al*. [17] involved 28 patients with confirmed ruptured globes and 28 in the control group. Although ACD measurements on CT scans were not possible for all OGI cases (15 eyes), a difference in ACD >0.4 mm between the two eyes was identified as an excellent factor. This criterion had a specificity of 100% and a sensitivity of 73% in detecting OGIs on CT scans. The differences in ACD between the ruptured and non-ruptured groups were statistically significant (*P* = 0.001). Specifically, 15 cases of globe rupture showed decreased ACD on the affected side, whereas two cases had increased ACD in the affected eye. ACD was measured using a radiological caliber from the back of the cornea to the front surface of the lens at the equatorial level. This study concluded that the single most accurate CT finding for diagnosing an OGI was ACD asymmetry.

### The association between open globe injury and concurrent orbital wall fracture

One study by Chieh Chou *et al*. [16] examined the accuracy of CT scans in detecting a ruptured globe in patients with concurrent orbital wall fractures. The study included 136 patients (136 eyes) with a history of facial trauma, all of whom underwent orbital CT imaging. Among them, 32 eyes (26.47%) had a ruptured globe upon clinical examination, whereas 41 eyes (30%) showed orbital wall fractures. The relative risk of globe rupture among the different types of wall fractures was 0.692, 0.459, 2.286, and 0.637 for superior, inferior lateral, and medial wall fractures, respectively, with 95% confidence intervals. These findings suggest that lateral walls increase the risk of concurrent globe rupture. Conversely, medial and inferior orbital wall fractures tend to protect against a ruptured globe.

### The ability of CT scans to predict visual prognosis in patients with open globe injuries

A study by Joseph *et al*. [19] evaluated 142 patients who underwent CT imaging for traumatic eye injuries, including 80 patients with confirmed open globe injuries. The study found that patients with poor visual outcomes—defined as visual acuity of ≤5/200 or who underwent enucleation—were more likely to exhibit detectable abnormalities on CT scans compared to those with better visual prognoses. Moreover, most eyes not identified by CT as having an OGI retained good visual acuity (≥20/40) in 9 out of 12 cases. The features associated with poor outcomes were moderate to severe scleral deformity, vitreous hemorrhage, lens absence, and distortion of the vitreous cavity. The mean number of CT abnormalities observed in patients varied based on their final visual acuity, with 2.1 findings in those with 20/40 or better, 4.9 in those with worse than 5/200, and 7.4 in cases requiring enucleation. The sensitivity of CT scans for detecting OGIs differed by injury location, with 61% for corneal OGIs, 82% for scleral OGIs, and 80% for combined corneal-scleral OGIs.

### The difference between axial and coronal CT scan cuts

In a study by Arabi *et al*. [20] investigating 133 eyes of 133 patients with ocular trauma, 61 had an OG based on clinical or surgical evaluation. The sensitivity of axial CT scan cuts for detecting OGIs was 74%, with a specificity of 90%. The sensitivity for coronal CT scan cuts was 65%, with a specificity of 89%. Combining axial and coronal CT scan cuts improved sensitivity to 79% and specificity to 93%. Notably, the sensitivity of the axial cut was higher than that of the coronal cut in detecting OGIs located posteriorly in Zone III and caused by a sharp object (*P* = 0.012). In another study by Joseph *et al*. [19], a similar finding was reported, indicating that the sensitivity of combined axial and coronal cuts ranged from 70% to 86%.

### The role of CT scan in detecting occult open globe injuries

In a study by Arey *et al*. [22], 48 eyes of 46 patients underwent surgical exploration to rule out occult OGs, with 34 confirmed cases. The accumulated sensitivity, specificity, positive predictive value, and negative predictive value of CT for detecting occult OGIs were 71%, 79%, 89%, and 52%, respectively. Statistically significant CT findings in occult OGIs were change in globe contour (*P* = 0.001), volume loss (*P* = 0.003), lens dislocation or absence (*P* = 0.048), vitreous hemorrhage (*P* = 0.003), and retinal detachment (*P* = 0.044).

### Differences between radiologist and ophthalmologist interpretations of CT scans

This review identified five studies in which CT scans were interpreted solely by radiologists, while three studies involved both ophthalmologists and radiologists in the interpretation process. We applied the chi-square test to assess whether diagnostic accuracy differed between these two approaches, which yielded a *P* value of 0.332, indicating no statistically significant difference between the two groups. The study by Crowell *et al*. [21] was excluded from this calculation because it did not define combined accuracy indices for neuroradiologists and ophthalmologists.

**Figure 6:**
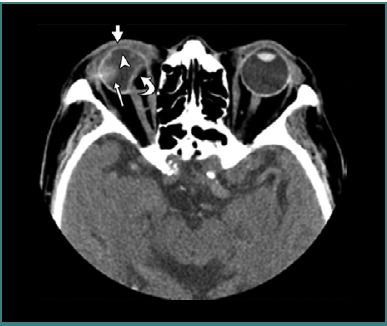
Axial computed tomography scan without contrast of a right open globe injury. The thick straight arrow indicates eyelid hematoma, the thin straight arrow represents vitreous hemorrhage, the arrowhead shows lens dislocation, and the curved arrow highlights scleral wall irregularities. Image reproduced with permission from the American Roentgen Ray Society: Yuan WH, Hsu HC, Lin YY, Liao SW. CT of globe rupture: analysis and frequency of findings. AJR Am J Roentgenol. 2014;202(5):4-5. ^©^ 2014 American Roentgen Ray Society.

**Figure 7:**
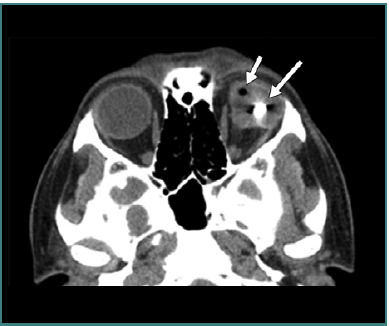
Axial computed tomography scan without contrast of left open globe. The small arrow represents intraocular air and the large arrow represents the metallic intraocular foreign body. Image reproduced with permission from the American Roentgen Ray Society: Yuan WH, Hsu HC, Lin YY, Liao SW. CT of globe rupture: analysis and frequency of findings. AJR Am J Roentgenol. 2014;202(5):4-5. ^©^ 2014 American Roentgen Ray Society.

**Figure 8:**
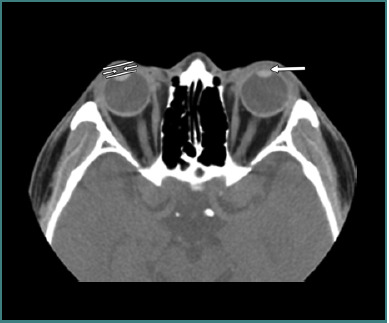
Axial computed tomography scan without contrast of left open globe. The single arrow represents shallow anterior chamber depth (ACD). The parallel lines represent the ACD measured perpendicular to the long axis of the lens, from the back surface of the cornea to the front surface of the lens (double arrows). In this case, the ACD difference was 2.2 mm. Image reproduced with permission from the American Roentgen Ray Society: Yuan WH, Hsu HC, Lin YY, Liao SW. CT of globe rupture: analysis and frequency of findings. AJR Am J Roentgenol. 2014;202(5):4-5. ^©^ 2014 American Roentgen Ray Society.

Figures 6, 7, and 8 (reprinted with permission from the American Roentgen Ray Society) illustrate examples of OGIs detected on CT scans, representing false positives and false negatives cases.

## DISCUSSION

OGIs are among the most devastating eye injuries, often resulting in significant visual and functional impairments and imposing social and economic burdens. CT is widely used for diagnosing OGIs. However, its accuracy and efficiency in detecting globe integrity after traumatic eye injury need comprehensive review. To the best of our knowledge, this is the first comprehensive review to investigate the accuracy of CT in identifying OGIs. In this review, we included nine observational studies that assessed the accuracy of CT scans in detecting OGIs. The sample size was 917 eyes of 883 patients. Most participants were men (77%), with a mean age of 38 years. The average sensitivity and specificity were 0.77 and 0.94, respectively. The overall diagnostic accuracy of CT in detecting OGIs based on the results of the area under the curve was 0.91.

A B-scan is recognized for its accuracy and utility in detecting additional ocular pathologies, such as retinal detachment and IOFBs, following the repair of OGIs. However, the role of the B-scan in suspected OGIs is uncommon. Using a B-scan in such cases may pose risks, including increased risk of extraocular extrusion of eye content, increased risk of artifacts in cases of disrupted eye contour, and elevated risk of infections during manipulation of non-surgically closed globes [24].

Among the six studies that mentioned the most common signs of OGIs on CT scans, scleral/globe deformity or wall irregularity was the most prevalent, observed in 139 of 191 cases (72.77%) [11,15,18,19,20,22]. This finding aligns with a study by Ameli *et al*. [25], which investigated the correlation between CT findings and the Ocular Trauma Score (OTS) in 182 eyes with OGIs. Their study found that the most common CT abnormalities were scleral irregularity or collapse of the globe, followed by altered vitreous density. The study observed that a higher number of CT findings were associated with a higher OTS. Additionally, it highlighted that the specific CT scan findings associated with higher OTS grades were changes in vitreous density, chorioretinal thickening, and the presence of intraocular air or foreign bodies.

In our search, two studies [19,20] confirmed the importance of employing combined axial and coronal CT scan cuts for detecting OGIs, leading to higher sensitivity. However, Arabi *et al*. [20] observed that although axial CT exhibited higher sensitivity than coronal CT, the difference (*P* = 0.07) was statistically insignificant between the two readings for Zone I and Zone II injuries. Conversely, in Zone III injuries, the axial CT view showed a statistically significant difference (*P* = 0.012).

This review revealed no statistical differences in sensitivity and specificity between the two groups (radiologists vs. radiologists and ophthalmologists) when interpreting CT scans.

With an AUC of 0.91, CT is a valuable diagnostic tool for suspected cases of OGI where clinical examination falls short. However, caution is warranted in interpreting negative results, particularly in scenarios suggesting an OGI based on the mechanism of injury. Early surgical intervention remains crucial to avert permanent vision loss [6].

### Limitations

This study has a few limitations. Firstly, the heterogeneity among the included studies regarding CT imaging protocols and settings may have influenced result visualization and interpretation. Secondly, the evaluation of CT scans by radiologists or a combination of ophthalmologists and radiologists varied across studies, necessitating uniformity among methodologies. Finally, the relatively small sample sizes in individual studies could have implications for the generalizability of the findings.

## CONCLUSION

CT provides high diagnostic accuracy for ocular injuries and is particularly useful when an open globe injury is clinically suspected. While the role of a CT scan is significant, caution is necessary for its interpretation. Surgical exploration should be considered if there is any doubt regarding the diagnosis. Further studies with larger cohorts and standardized protocols are essential to refine the diagnostic role of CT scans in eye trauma, particularly for serious eye injuries like OGI.

**Figure 9:**
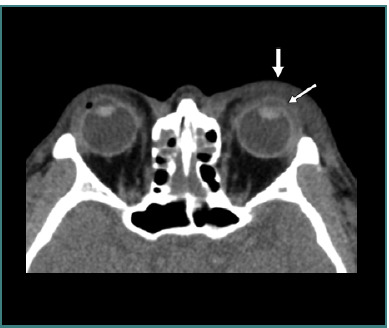
Axial computed tomography scan without contrast of left globe (false positive open globe). The thin arrow represents wall deformity (clinical examination showed subconjunctival hemorrhage and edema) and the thick arrow represents eyelid hematoma. Image reproduced with permission from the American Roentgen Ray Society: Yuan WH, Hsu HC, Lin YY, Liao SW. CT of globe rupture: analysis and frequency of findings. AJR Am J Roentgenol. 2014;202(5):4-5. ^©^ 2014 American Roentgen Ray Society.

**Figure 10:**
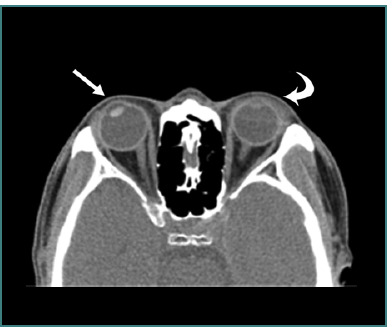
Axial computed tomography scan without contrast of left open globe (false negative). The curved arrow represents swelling of the left eyelid and the straight arrow represents normal anterior chamber depth (ACD) in the right eye compared to the shallow left eye. Image reproduced with permission from the American Roentgen Ray Society: Yuan WH, Hsu HC, Lin YY, Liao SW. CT of globe rupture: analysis and frequency of findings. AJR Am J Roentgenol. 2014;202(5):4-5. ^©^ 2014 American Roentgen Ray Society.

**Figure 11:**
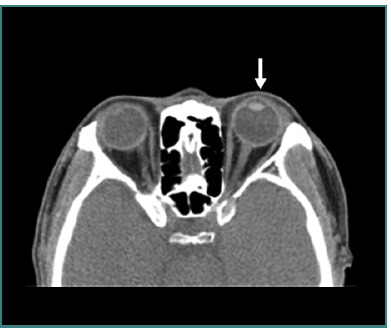
Axial computed tomography scan without contrast of left open globe (false negative). The arrow represents the missed shallow anterior chamber depth (ACD) of the left eye due to lens misalignment during the scan. Image reproduced with permission from the American Roentgen Ray Society: Yuan WH, Hsu HC, Lin YY, Liao SW. CT of globe rupture: analysis and frequency of findings. AJR Am J Roentgenol. 2014;202(5):4-5. ^©^ 2014 American Roentgen Ray Society.
